# Revolutionizing Neonatal Nutrition: Rethinking Definitions and Standards for Optimal Care

**DOI:** 10.1016/j.advnut.2024.100235

**Published:** 2024-04-26

**Authors:** Ariel A Salas

**Affiliations:** University of Alabama at Birmingham, Birmingham, AL, United States

In this issue of the Journal, Fenton et al. [1] offer an exemplary review of a crucial topic, shedding light on the interplay between prenatal factors leading to preterm birth and the subsequent growth trajectories of preterm and small infants. The review endorses a definition of "abnormal growth" using a fall below 2 SDs as a referent point. It also acknowledges the potential for catch-up growth in most preterm infants ≤36 mo of corrected age, highlights the limited utility of BMI monitoring in preterm infants, and provides recommendations for effective communication with parents regarding growth parameters.

Three other noteworthy topics that could be easily implemented in clinical practice deserve further elaboration. First, defining an acceptable amount of postnatal weight loss using SDs or *z*-scores holds significant implications. Current clinical evidence consistently indicates that an average decrease of 0.8 in weight-for-age *z*-scores is expected across different gestational age groups of preterm infants [2]. Therefore, weight loss > 0.8 or 1 during the first 2 wk after birth should be monitored closely to define individual expectations, particularly in small-for-gestational-age infants. Further weight loss in small-for-gestational-age infants could represent lean mass loss, given their reduced capacity for water retention. With the increasing availability of growth charts in electronic medical record systems, monitoring excessive weight loss using changes in *z*-scores could be more reliable than using weight loss percentages [3]. Monitoring this type of postnatal weight loss in clinical settings could validate or redefine the concept of preterm malnutrition [4], reducing variability and bias in the analysis of weight gain velocities.

Second, the call for the development of individualized growth trajectories due to multicausality in growth outcomes is insightful. The multifactorial nature of growth, including genetic differences and neonatal morbidities, underscores the importance of adequate nutrition and highlights the need to consider other influencing factors. Although size at birth can be attributed to a specific prenatal environment, genetic factors also play a significant role. Therefore, using growth references from diverse populations is essential when assessing preterm growth [5]. These individualized growth patterns should not be expected to follow growth curves because fluctuations, particularly in length and head circumference gains, are to be expected. To achieve this individualized goal, it is imperative to enhance the reliability of length measurements at birth in critically ill preterm infants, or explore other time points for measurement. Standardizing measurement practices is key to reducing biases and gaining clarity on how critical illness impacts growth trajectories [6].

Third, regarding body composition measurements, Fenton et al. [1] raise pertinent questions about current reference standards, suggesting the need to compare infants with similar postnatal ages. Comparing measurements obtained at term-equivalent age among preterm infants with measurements obtained shortly after birth in term infants creates an artificial idea that preterm infants have more body fat than term infants. However, comparing these same measurements to infants born at term with similar postnatal age in days may not yield statistically significant differences. Temporal differences in body fat percentage between preterm and term infants underscore the complexity of assessing body composition in this population and emphasize the need for longitudinal assessments [7].

In summary, this review significantly contributes to our understanding of growth patterns in preterm infants and provides valuable guidance to rethink definitions and standards for optimal nutritional care.

## Conflict of interest

AAS has received honoraria from the Lockwood Group for participation in Mead Johnson Nutrition advisory board meetings and has patented an instrumented feeding bottle.
